# Prostate Health Index and Prostate Health Index Density as Diagnostic Tools for Improved Prostate Cancer Detection

**DOI:** 10.1155/2020/9872146

**Published:** 2020-07-21

**Authors:** Marija Barisiene, Arnas Bakavicius, Diana Stanciute, Jolita Jurkeviciene, Arunas Zelvys, Albertas Ulys, Dalius Vitkus, Feliksas Jankevicius

**Affiliations:** ^1^Institute of Clinical Medicine, Faculty of Medicine, Vilnius University, Vilnius 03101, Lithuania; ^2^Laboratory of Molecular Oncology, National Cancer Institute, Vilnius 08660, Lithuania; ^3^Faculty of Health Sciences, Klaipeda University, Klaipeda 92294, Lithuania; ^4^Centre of Laboratory Medicine, Vilnius University Hospital Santaros Klinikos, Vilnius 08661, Lithuania

## Abstract

**Background:**

To evaluate the diagnostic potential of [-2] proPSA (p2PSA), %p2PSA, Prostate Health Index (phi), and phi density (PHID) as independent biomarkers and in composition of multivariable models in predicting high-grade prostatic intraepithelial neoplasia (HGPIN) and overall and clinically significant prostate cancer (PCa).

**Methods:**

210 males scheduled for prostate biopsy with total PSA (tPSA) range 2-10 ng/mL and normal digital rectal examination were enrolled in the prospective study. Blood samples to measure tPSA, free PSA (fPSA), and p2PSA were collected immediately before 12-core prostate biopsy. Clinically significant PCa definition was based on Epstein's criteria or ISUP grade ≥ 2 at biopsy.

**Results:**

PCa has been diagnosed in 112 (53.3%) patients. Epstein significant and ISUP grade ≥ 2 PCa have been identified in 81 (72.3%) and 40 (35.7%) patients, respectively. Isolated HGPIN at biopsy have been identified in 24 (11.4%) patients. Higher p2PSA and its derivative mean values were associated with PCa. At 90% sensitivity, PHID with cut-off value of 0.54 have demonstrated the highest sensitivity of 35.7% for overall PCa detection, so PHID and phi with cut-off values of 33.2 and 0.63 have demonstrated the specificity of 34.7% and 34.1% for ISUP grade ≥ 2 PCa detection at biopsy, respectively. In univariate ROC analysis, PHID with AUC of 0.77 and 0.80 was the most accurate predictor of overall and Epstein significant PCa, respectively, so phi with AUC of 0.77 was the most accurate predictor of ISUP grade ≥ 2 PCa at biopsy. In multivariate logistic regression analysis, phi improved diagnostic accuracy of multivariable models by 5% in predicting ISUP grade ≥ 2 PCa.

**Conclusions:**

PHID and phi have shown the greatest specificity at 90% sensitivity in predicting overall and clinically significant PCa and would lead to significantly avoid unnecessary biopsies. PHID is the most accurate predictor of overall and Epstein significant PCa, so phi is the most accurate predictor of ISUP grade ≥ 2 PCa. phi significantly improves the diagnostic accuracy of multivariable models in predicting ISUP grade ≥ 2 PCa.

## 1. Introduction

Prostate cancer (PCa) is the second most common cancer in males worldwide accounting for 13.5% of all new cancer cases and the sixth leading cause of cancer-related death among males [1]. Australia and New Zealand, so the Northern and Western European countries, belong to the highest incidence rate regions in the world (age standardized rate (ASR) per 100,000 of 86.4, 85.7, and 75.8, respectively), largely due to widespread use of prostate-specific antigen (PSA) testing and subsequent prostate biopsies (PB) [1, 2]. PCa is the 3rd most commonly diagnosed cancer by cancer site and the most common cancer among males in Lithuania with ASR of 70.2 per 100,000 males. It is the third most common cancer-related death among Lithuanian males (ASR: 11.3 per 100,000) [1].

Due to low PSA specificity in determining PCa, especially with the total PSA (tPSA) level below 10 ng/mL, the risk of PCa in males with tPSA between 4.1and 9.9 ng/mL and negative digital rectal examination (DRE) is about 20% with 85% probability, respectively, that these cancers would be organ confined [3]. On the other hand, some males may harbor PCa despite very low tPSA levels (<2.0 ng/mL) [4]. In up to 25% of all PB, premalignant condition, namely, high-grade prostatic intraepithelial neoplasia (HGPIN), is diagnosed [5, 6]. Of all screen-detected PCa based on tPSA screening programs, 42% are clinically insignificant, which means that the disease would never lead to clinical symptoms or death [7]. In turn, detection of clinically insignificant PCa consequently leads to overtreatment with its potential side effect profile or may cause an anxiety related to active surveillance strategy, so deterioration of quality of life in any scenario is inevitable in aging society. Thus, the decision to perform a PB with intent to detect clinically significant PCa, which indeed needs treatment, in males with tPSA levels within the “grey” zone is one of the main concerns in daily practice.

To overcome the limitations of tPSA, p2PSA derivatives, such as Prostate Health Index (phi) and percentage of p2PSA (%p2PSA), which combine the results of quantitative kallikrein immunoassays into a single numerical score, have been suggested. %p2PSA and phi have been associated with improved overall and aggressive PCa detection over tPSA and fPSA/tPSA ratio (%fPSA) in several studies [8–11]. The objective of the present study was to evaluate the diagnostic potential of p2PSA, %p2PSA, phi, and phi density (PHID) as independent biomarkers and in combination with other demographic and clinical parameters, to predict overall and clinically significant PCa. The ability of p2PSA and its derivatives to discriminate HGPIN at biopsy was evaluated.

## 2. Materials and Methods

### 2.1. Patients and Samples

A prospective clinical trial was initiated at Vilnius University Hospital Santaros Klinikos (Lithuania) and National Cancer Institute (Lithuania) from January 2015 till December 2016. Males older than 50 years old with tPSA range from 2 to 10 ng/mL and normal DRE referred for PB were included into the study. Previous diagnosis of PCa, history of open or endoscopic prostate surgery, PB within 3 months prior the study, usage of 5-alpha-reductase inhibitors, active urinary tract infection, and acute prostatitis were considered exclusion criteria. The study was approved by the Regional Biomedical Research Ethics Committee (Nr 158200-14-759-273), and written informed consent was obtained from all patients.

Blood samples to measure tPSA, fPSA, and p2PSA were collected immediately before PB for every patient and processed within 3 hours after the collection due to instability of p2PSA at room temperature [12]. The samples were stored at -80°C before testing in a single laboratory using the Beckman Coulter Access® 2 Immunoassay Analyzer and Access Hybritech® reagents and calibrators for all assays, including tPSA, fPSA, and p2PSA. Hybritech calibration was used for tPSA and fPSA. %p2PSA was calculated using formula (p2PSA/free PSA (fPSA)) × 100 and phi as (p2PSA/fPSA) × √tPSA [13].

Transrectal ultrasound was used to measure prostate. Prostate volume (PV) was calculated using formula prostate length × height × width × 0.52. PSA density (PSAD) and PHID were calculated as tPSA and phi divided by the volume of the prostate. PB was performed by transrectal approach using the standardized 12-core random sampling protocol. PB specimens were evaluated at National Centre of Pathology (Lithuania) by dedicated pathologists blinded to the blood serum results. Gleason score was evaluated according to the 2005 Guidelines of International Society of Urological Pathology (ISUP), and ISUP grades were assigned according to ISUP 2014 recommendations [14]. Clinically significant PCa was defined as having met the clinically significant PCa definition according to Epstein's criteria (PSA density ≥ 0.15 ng/mL/g, Gleason score ≥ 7, ≥3 positive cores for PCa, and presence of ≥50% of PCa per any core) [15] or if ISUP grade ≥ 2 has been identified at biopsy.

### 2.2. Statistical Analysis

Descriptive statistics was used to characterize patients in groups according to biopsy results. The Shapiro-Wilk test was used to determine the normality of the variables. Student's *t*-test, Wilcoxon singed rank test, and Pearson's chi-squared tests were used for comparisons of continuous and qualitative variables, respectively. The relationship between p2PSA, %p2PSA, phi, and biopsy Gleason score was evaluated using the Spearman correlation analysis. Before the logistic regression analysis, Spearman correlations were used to check the correlation between the variables. Univariate and multivariate binary logistic regression models have been concluded for the prediction of PCa. The multivariate logistic regression models were fitted using the forward stepwise approach. With the intention to find out if a newer biomarker and its indices can improve the diagnostic accuracy of logistic regression models, p2PSA, %p2PSA, and phi were added to the base model composed of repeated PB, PV, fPSA, and %fPSA. The accuracy of the tests was measured by the area under the receiver operating characteristic (ROC) curves (AUC). Odds ratios and 95% confidence intervals (CI) have been calculated. The specificity at fixed 90% sensitivity, as well as the best combination of sensitivity and specificity, and the positive (PPV) and negative predictive values (NPV) have been estimated. The determination of cut-off values was based on Youden's index. DeLong et al.'s method has been used to compare the ROC curves [16]. Decision curve analysis (DCA) [17] was used to determine the net benefit of single biomarkers in guiding clinical decision-making on PB. Statistical analysis was performed using SAS version 9.2 (SAS Institute Inc., Carry, NC, USA). *P* value < 0.05 was defined as statistically significant.

## 3. Results

PCa has been diagnosed in 112 (53.3%) out of 210 males enrolled into the study. Clinicopathological characteristics of the study cohort are summarized in Table 1. Clinically significant PCa according to Epstein's criteria and ISUP grade ≥ 2 have been identified in 81 (72.3%) and 40 (35.7%) out of 112 patients, respectively. Isolated HGPIN at biopsy has been identified in 24 (11.4%) patients. Overall and clinically significant PCa, as well as ISUP < 2 PCa, was diagnosed more frequently during the first PB than in the repeated PB setting (89.3% vs. 10.7%, 90.1% vs. 9.9%, 90.0% vs. 10.0%, and 88.9% vs. 11.1%, respectively; all *P* < 0.05; Table 1). PV has been found to be significantly smaller in patients harboring overall PCa, as well as in patients with Epstein significant and ISUPgrade ≥ 2PCa in comparison to patients in the non-PCa group (38.55 mL, 36.16 mL, and 37.78 mL vs. 55.02 mL, respectively) or to patients with isolated HGPIN at biopsy (38.55 mL, 36.16 mL, and 37.78 mL vs. 52.80 mL, respectively; all *P* < 0.05; Table 1). tPSA mean value was significantly slightly higher only in patients with Epstein significant PCa in comparison to patients in the non-PCa group (4.85 ng/mL vs. 4.11 ng/mL, respectively; *P* = 0.004; Table 1). PSAD mean value was higher in patients with overall, Epstein significant, and ISUP grade ≥ 2 PCa in comparison to patients in the non-PCa group or with isolated HGPIN at biopsy (0.14 ng/mL/cc, 0.16 ng/mL/cc, and 0.13 ng/mL/cc vs. 0.09 ng/mL/cc, respectively; all *P* < 0.01; Table 1).

fPSA and %fPSA mean values were significantly lower, so phi and PHID mean values were higher in patients with overall, Epstein significant, and ISUP grade ≥ 2 PCa than in patients in the non-PCa group and isolated HGPIN at biopsy (0.60 ng/mL, 0.59 ng/mL, and 0.57 ng/mL vs. 0.72 ng/mL and 0.80 ng/mL, so 48.31, 52.26, and 55.62 vs. 35.62 and 38.05, respectively; all *P* < 0.05; Table 1). %p2PSA mean values were higher in patients with overall PCa in comparison to patients in the non-PCa group (2.34 vs. 1.83, *P* < 0.001), as well as in patients with Epstein significant andISUP ≥ 2PCa in comparison to patients in the non-PCa group and isolated HGPIN at biopsy (2.44 and 2.62 vs. 1.83 and 1.93, respectively; *P* < 0.05; Table 1).

Significant correlations were revealed between biopsy ISUP grade ≥ 2 and %p2PSA (*ρ* = 0.30, *P* < 0.001), phi (*ρ* = 0.36, *P* < 0.001), and PHID (*ρ* = 0.42, *P* < 0.001).

Using Youden's index, PHID with cut-off value of 1.04 for detection of overall PCa, 1.06 for Epstein significant PCa, and 1.04 for ISUP grade ≥ 2 PCa have outperformed tPSA, PSAD, fPSA, %fPSA, p2PSA, %p2PSA, and phi and showed the best diagnostic power, with sensitivity and specificity of 61.6% and 81.6%, 71.6% and 78.3%, and 75.0% and 66.5%, respectively (Tables 2–4).

At 90% sensitivity for detecting overall PCa, PHID with cut-off value of 0.54 has had the specificity of 35.7%, which was higher than other biomarkers. At 90% sensitivity for detecting Epstein significant PCa, PSAD with cut-off value of 0.07 ng/mL/cc has shown the specificity of 41.9% that was slightly higher than phi and PHID (35.7% and 36.4%, respectively). However, at 90% sensitivity for detecting ISUP grade ≥ 2 PCa, phi and PHID with cut-off values of 33.2 and 0.63 have shown the highest specificity of 34.7% and 34.1%, respectively (Tables 2–4).

At 90% sensitivity for detecting overall PCa, PHID would lead to avoid 21.4% of prostate biopsies in comparison to 16.2% for phi and PSAD, 19% for %p2PSA, 10.5% for tPSA, and less than 7% for the rest of the biomarkers. At 90% sensitivity for detecting ISUP grade ≥ 2 PCa, phi and PHID, as well as PSAD for detecting Epstein significant PCa, would lead to avoid 30% of prostate biopsies.

**Figure 1:**
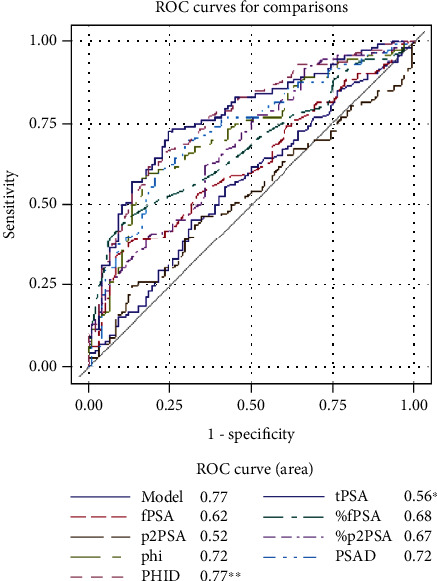
Receiver operating characteristic curves representing the diagnostic ability of blood serum biomarkers in predicting overall prostate cancer. Abbreviations: ISUP: International Society of Urological Pathology; fPSA: free prostate-specific antigen; %fPSA: free to total PSA ratio; phi: Prostate Health Index; PHID: phi density; PSAD: PSA density; ROC: receiver operating characteristic; tPSA: total PSA; %p2PSA: p2PSA to fPSA ratio. All significant differences are marked with asterisk (^∗^) and (^∗∗^): ^∗^*P* < 0.05 for tPSA vs. p2PSA, phi, %fPSA, and PSAD. ^∗∗^*P* < 0.05 for PHID vs. tPSA, fPSA, %fPSA, p2PSA, %p2PSA, and phi.

**Figure 2:**
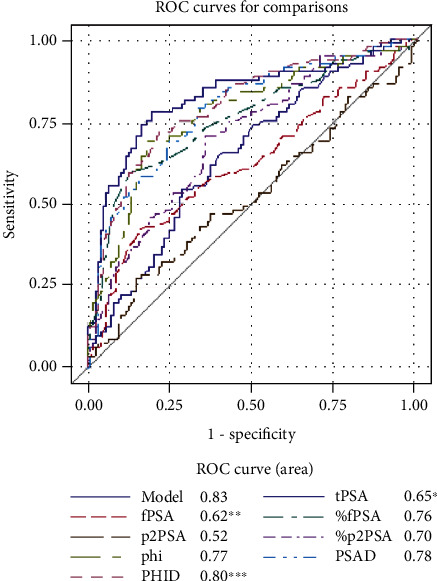
Receiver operating characteristic curves representing the diagnostic ability of blood serum biomarkers in predicting clinically significant prostate cancer according to Epstein's criteria. Abbreviations: ISUP: International Society of Urological Pathology; fPSA: free prostate-specific antigen; %fPSA: free to total PSA ratio; phi: Prostate Health Index; PHID: phi density; PSAD: PSA density; ROC: receiver operating characteristic; tPSA: total PSA; %p2PSA: p2PSA to fPSA ratio. All significant differences are marked with asterisk (^∗^), (^∗∗^), and (^∗∗∗^): ^∗^*P* < 0.05 for tPSA vs. %fPSA, p2PSA, phi, PSAD, and PHID. ^∗∗^*P* < 0.05 for fPSA vs. %fPSA, phi, PSAD, and PHID. ^∗∗∗^*P* < 0.05 for PHID vs. p2PSA and %p2PSA.

**Figure 3:**
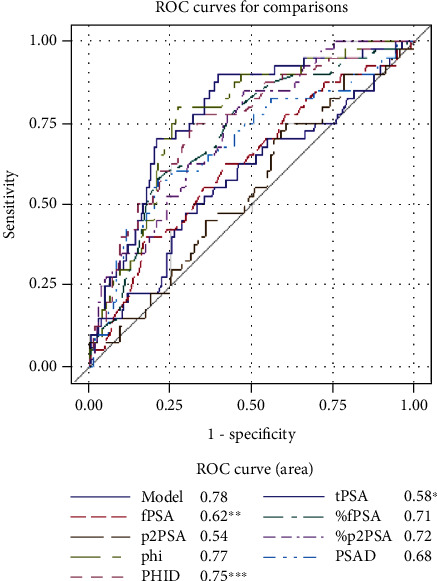
Receiver operating characteristic curves representing the diagnostic ability of blood serum biomarkers in predicting ISUP grade ≥ 2 prostate cancer. Abbreviations: ISUP: International Society of Urological Pathology; fPSA: free prostate-specific antigen; %fPSA: free to total PSA ratio; phi: Prostate Health Index; PHID: phi density; PSAD: PSA density; ROC: receiver operating characteristic; tPSA: total PSA; %p2PSA: p2PSA to fPSA ratio. All significant differences are marked with asterisk (^∗^), (^∗∗^), and (^∗∗∗^): ^∗^*P* < 0.05 for tPSA vs. %fPSA, phi, PSAD, and PHID. ^∗∗^*P* < 0.05 for fPSA vs. %fPSA, %p2PSA, phi, and PHID. ^∗∗∗^*P* < 0.05 for PHID vs. p2PSA and PSAD.

In univariate ROC analysis (Table 5), PHID with AUC of 0.77 was the most accurate predictor of overall PCa significantly outperforming tPSA, fPSA, %fPSA, p2PSA, %p2PSA, and phi (all *P* < 0.05; see Figure 1). PHID was the most accurate predictor of Epstein significant PCa with AUC of 0.80 outperforming tPSA, fPSA, p2PSA, and %p2PSA (all *P* < 0.05; see Figure 2). However, phi was the most accurate predictor of ISUP grade ≥ 2 PCa at biopsy with AUC of 0.77 significantly outperforming tPSA and fPSA (all *P* < 0.05; see Figure 3).

In multivariate logistic regression analysis by adding p2PSA and its derivatives one by one to the base logistic regression model, which consisted of repeated biopsy, PV, fPSA, and %fPSA variables, it has been estimated that PHID is the most significant predictor for overall PCa (OR 4.34,*P* < 0.001), Epstein significant PCa (OR 3.58,*P* < 0.001), and ISUPgrade ≥ 2PCa (OR 2.38,*P* < 0.001). In all multivariate logistic regression model analysis, p2PSA, %p2PSA, phi, and PHID have achieved an independent predictor status. The only phi added to the base multivariate logistic regression model significantly improved diagnostic accuracy by 5% in predicting ISUP grade ≥ 2 PCa at biopsy (AUC 0.74 and 0.79, respectively; *P* = 0.039; Table 6).

**Figure 4:**
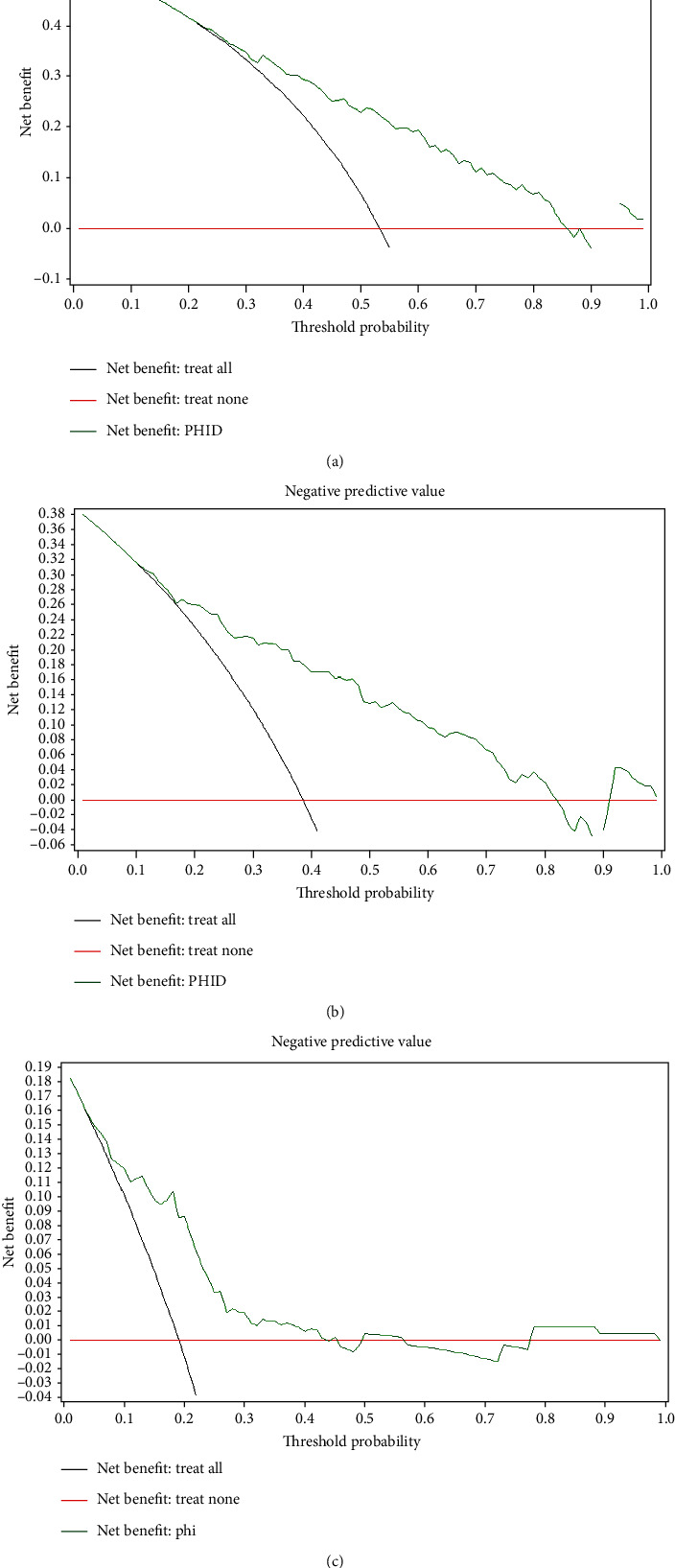
Decision curve analysis for prostate cancer biomarkers that have demonstrated the best net benefit (a) for prediction of overall, (b) for prediction of Epstein significant, and (c) for prediction of ISUP ≥2 prostate cancer. The net benefit is plotted against the threshold probability. The unit of net benefit is true positives. Abbreviations: phi: Prostate Health Index; PHID: phi density.

-.09pt?>We performed DCA to determine the net benefit for each biomarker in predicting overall and clinically significant PCa. The best net benefit was determined for PHID in predicting overall and Epstein significant PCa (Figures 4(a) and 4(b)) and for phi in predicting ISUP grade ≥ 2 PCa at biopsy (Figure 4(c)). At 20% threshold probability, based on PHID, 45 and 26 of 100 biopsied patients would be diagnosed overall and Epstein significant PCa, respectively, so based on phi,ISUP ≥ 2PCa would be diagnosed in 9 of 100 biopsied males.

## 4. Discussion

In today's clinical practice, there is no universal definition of clinically significant PCa. According to our results, clinically significant PCa according to Epstein's criteria have been diagnosed to 72% of patients and PCa harboring ISUP grade ≥ 2 to 36% of patients. Therefore, the decision to perform PB based on a single serum biomarker with intent to detect clinically significant disease is still a challenge in urological practice.

Due to the limited specificity of tPSA, there is considerable interest in new diagnostic biomarkers for PCa that could overcome tPSA limitations and demonstrate improved specificity. It was found that precursor forms of PSA constitute the predominant fraction of fPSA in PCa serum [18]. Histological analyses of prostate specimens have shown that primarily precursor of PSA, called p2PSA, is elevated in the peripheral zone, while it was undetectable in the transition zone, leading to the consensus that this isoform is more cancer specific than tPSA [19]. Subsequently, it was found that p2PSA isoform is providing higher concentration levels in PCa patients' blood serum [20]. Recently, it was also revealed that p2PSA could be a marker for PCa aggressiveness already several years before diagnosis [21]. So p2PSA derivatives, such as phi and %p2PSA, have been suggested for PCa diagnostics with intent to increase the specificity of tPSA [13]. Indeed, our study has demonstrated that %p2PSA and phi are associated with ISUP grade ≥ 2 disease and could be used for PCa detection, which was also confirmed by other authors [8, 22, 23]. It is reported that %p2PSA and phi mean values are significantly higher not only in PCa patients in comparison to non-PCa patients, but the difference is found between PCa patients and patients with isolated HGPIN at biopsy [6]. In our study, isolated HGPIN at biopsy have been identified in 11.4% of patients, and likewise noted above, we determined significantly higher not only %p2PSA and phi but also PHID mean values in patients with overall, Epstein significant, and ISUP grade ≥ 2 PCa in comparison with patients with isolated HGPIN at biopsy (Table 1).

We estimated that higher PHID mean values are associated not only with overall but also with clinically significant PCa (Table 1), which is consistent with previous studies [24, 25].

According to our study results, the specificity of 35.7% at 90% sensitivity demonstrated the advantages for PHID at cut-off value of 0.54 in comparison with all other investigated biomarkers for overall PCa detection (Table 2). Our results are consistent with previous studies, when PHID at a cut-off of 0.49 and 0.43 at 90.7% and 97.9% sensitivity, respectively, demonstrated the specificity of 30% and 38% for detection of overall and clinically significant PCa (i.e., ISUP grade ≥ 2 PCa or Gleason score 3 + 3 cancer detected in >2 cores or >50% of any one core) [24, 25].

At 90% sensitivity, to detect Epstein significant PCa, phi with cut-off of 31.92 and PHID with cut-off of 0.61 have shown the specificity of 35.7% and 36.4%, respectively, which was slightly inferior to PSAD with cut-off of 0.07 ng/mL/cc and specificity of 41.9% (Table 3). However, at 90% sensitivity, to detect ISUP grade ≥ 2 PCa, phi and PHID with cut-off values of 33.2 and 0.63 have shown the highest specificity of 34.7% and 34.1%, respectively (Table 4). According to the literature, the specificity between 29.7% and 45.2% at 90% sensitivity for phi outperformed the specificity of tPSA (7.8-26.4%) and %fPSA (28.5%) to detect ISUP grade ≥ 2 PCa [8, 10, 26, 27].

At 90% sensitivity, for detecting overall PCa, PHID would lead to avoid 21.4% of prostate biopsies in comparison to 16.2% for phi and PSAD, 19% for %p2PSA, 10.5% for tPSA, and less than 7% for the rest of the biomarkers. At 90% sensitivity for detecting ISUP grade ≥ 2 PCa, phi and PHID, as well as PSAD for detecting Epstein significant PCa, would lead to avoid 30% of prostate biopsies.

On univariate ROC analysis, we have identified PHID as a more accurate predictor for overall PCa detection in comparison to tPSA, fPSA, %fPSA, p2PSA, %p2PSA, and phi (all *P* < 0.05; see Figure 1). What is more important, we came to a conclusion that PHID is the most accurate predictor of Epstein significant PCa with AUC of 0.80 outperforming tPSA, fPSA, p2PSA, and %p2PSA (all *P* < 0.05; see Table 2). However, phi was the most accurate predictor of ISUP grade ≥ 2 PCa at biopsy with AUC of 0.77 significantly outperforming tPSA and fPSA (all *P* < 0.05; see Figure 3). Other authors have reported results that are in agreement with our findings, where PHID significantly outperformed tPSA, fPSA, and %fPSA in prediction for overall PCa, so PHID and phi had the greatest predictive accuracy for clinically significant prostate cancer [25, 26].

However, there is no ideal single biomarker and a multivariable approach for improved PCa detection is advocated [28]. The multivariate logistic regression models for PCa prediction, which include p2PSA and its derivatives, are described in many clinical studies. It was estimated that addition of p2PSA derivatives in a multivariate logistic regression model, which consisted of the most common demographic and clinical PCa predictors, has improved predictive accuracy for overall PCa detection up to 11% and outperformed its independent components [22, 23, 29, 30]. Recently, Loeb et al. came to a conclusion that inclusion of phi into the multivariate logistic regression model, which consisted of age, previous biopsy, PV, and tPSA, improved AUC from 0.70 to 0.75 to predict ISUP grade ≥ 2 PCa in males with negative DRE and PSA between 2 and 10 ng/mL [31]. In our study, we have revealed that only phi inclusion into the multivariate logistic regression model, which consisted of previous biopsy, PV, fPSA, and %fPSA, has improved AUC to predict ISUP grade ≥ 2 PCa from 0.74 to 0.79 (*P* = 0.04).

Summarizing the available scientific data, it is concluded that phi and PHID could help to improve individual risk assessment for early particularly clinically significant PCa detection, to reduce unnecessary biopsies, either may help to select patients eligible for active surveillance and may play a role in treatment decision-making [32].

Nevertheless, we should address several limitations of the present study. Firstly, a small study cohort could lead to inability to establish cut-off values that could be useful in daily practice. Secondly, it was not possible to make a comparative analysis with other commercially available blood biomarkers, including 4K test and PCA3, which could be useful tools in making decisions on PB. Thirdly, multiparametric magnetic resonance imaging, widely used nowadays in clinical practice, was not included in our protocol. Finally, several dedicated pathologists have been involved which could make a bias in pathological analysis.

In spite of these limitations, there is a certain strength in our study. This is a prospective study in which all males underwent PB under a standardized protocol. The study population consisted of males with tPSA levels within the “grey” zone and negative DRE, representing the most debatable group of population in making decisions on PB.

## 5. Conclusions

PHID and phi have shown the greatest specificity at 90% sensitivity in predicting overall and ISUP grade ≥ 2 PCa. phi and PHID have shown slightly inferior specificity at 90% sensitivity in comparison with PSAD in predicting Epstein significant PCa. phi and PHID, as well as PSAD, could lead to avoid 30% of unnecessary prostate biopsies in everyday clinical practice. PHID is the most accurate predictor of overall and Epstein significant PCa, so phi is the most accurate predictor of ISUP grade ≥ 2 PCa. phi significantly improves the diagnostic accuracy of multivariable models in predicting ISUP grade ≥ 2 PCa at biopsy.

## Figures and Tables

**Table 1:** Clinicopathological characteristics of the study cohort.

					PCa cases (*N* = 112)			
					Epstein's criteria		ISUP grade	
	All cohort	Benign cases	HGPIN at biopsy	PCa cases	NCS PCa	CS PCa	<2	≥2
Patients, *N* (%)	210 (100)	98 (46.7)	24 (11.4)	112 (53.3)	31 (27.7)	81 (72.3)	72 (64.3)	40 (35.7)
Age (years)								
Median	62	63	66	62	57	62	60.5	62.5
Mean ± SD	63 ± 7.09	63.71 ± 6.9	66.00 ± 6.96	62.42 ± 7.22^#^	59.74 ± 6.83^∗^^#^	63.44 ± 7.14	61.64 ± 7.3^∗^^#^	63.83 ± 6.94
PV (mL)								
Median	43	50	50	34	47	32	37	33
Mean ± SD	46.20 ± 22.47	55.02 ± 25.39	52.80 ± 21.75	38.55 ± 16.13^∗^^#^	44.81 ± 14.71	36.16 ± 16.1^∗^^#^	38.99 ± 14.58^∗^^#^	37.78 ± 18.77^∗^^#^
Biopsy:								
Primary, *n* (%)	172 (81.9)	72 (73.5)	17 (70.8)	100 (89.3)^∗∗^	27 (87.1)	73 (90.1)^∗∗^	64 (88.9)^∗∗^	36 (90)^∗∗^
Repeated, *n* (%)	38 (18.1)	26 (26.5)	7 (29.2)	12 (10.7)	4 (12.9)	8 (9.9)	8 (11.1)	4 (10)
tPSA (ng/mL)								
Median	3.90	3.58	3.81	3.99	3.17	4.39	3.94	4.34
Mean ± SD	4.32 ± 1.83	4.11 ± 1.70	4.13 ± 1.55	4.49 ± 1.91	3.55 ± 1.32	4.85 ± 2.00^∗^	4.29 ± 1.69	4.86 ± 2.25
PSAD (ng/mL/cc)								
Median	0.10	0.08	0.08	0.11	0.08	0.13	0.10	0.13
Mean ± SD	0.11 ± 0.07	0.09 ± 0.06	0.09 ± 0.06	0.14 ± 0.08^∗^^#^	0.09 ± 0.03	0.16 ± 0.08^∗^^#^	0.13 ± 0.07^∗^^#^	0.15 ± 0.08^∗^^#^
fPSA (ng/mL)								
Median	0.59	0.63	0.69	0.55	0.58	0.50	0.56	0.50
Mean ± SD	0.66 ± 0.33	0.72 ± 0.33	0.80 ± 0.40	0.60 ± 0.32^∗^^#^	0.63 ± 0.31	0.59 ± 0.32^∗^^#^	0.62 ± 0.32^∗^	0.57 ± 0.32^∗^^#^
%fPSA								
Median	15.50	17.00	18.00	13.00	18.00	11.00	15.50	10.50
Mean ± SD	16.00 ± 6.82	18.10 ± 6.49	19.10 ± 6.54	14.10 ± 6.58^∗^^#^	18.00 ± 5.99	12.61 ± 6.21^∗^^#^	15.14 ± 6.89^∗^^#^	12.23 ± 5.60^∗^^#^
p2PSA (pg/mL)								
Median	11.36	11.16	11.13	11.48	11.61	11.37	11.57	11.41
Mean ± SD	12.78 ± 6.96	12.24 ± 5.71	13.61 ± 6.18	13.25 ± 7.88	12.44 ± 5.91	13.56 ± 8.53	12.63 ± 6.36	14.36 ± 10.05
%p2PSA								
Median	2.02	1.82	2.00	2.21	1.89	2.29	2.10	2.39
Mean ± SD	2.10 ± 0.80	1.83 ± 0.62	1.93 ± 0.63	2.34 ± 0.86^∗^	2.08 ± 0.71	2.44 ± 0.89^∗^^#^	2.18 ± 0.78^∗^	2.62 ± 0.94^∗^^#^
phi								
Median	40.19	35.03	36.85	46.36	36.4	49.74	42.91	51.62
Mean ± SD	42.39 ± 17.80	35.62 ± 12.58	38.05 ± 13.03	48.31 ± 19.55^∗^^#^	37.97 ± 11.57	52.26 ± 20.56^∗^^#^	44.24 ± 16.70^∗^	55.62 ± 22.24^∗^^#^
PHID								
Median	0.98	0.65	0.80	1.31	0.85	1.51	1.13	1.59
Mean ± SD	1.18 ± 0.80	0.79 ± 0.53	0.88 ± 0.56	1.56 ± 1.07^∗^^#^	0.99 ± 0.58^∗^	1.772 ± 1.13^∗^^#^	1.38 ± 0.89^∗^^#^	1.88 ± 1.28^∗^^#^

Abbreviations: CS: clinically significant; fPSA: free prostate-specific antigen; %fPSA: free to total PSA ratio; HGPIN: high-grade prostatic intraepithelial neoplasia; ISUP: International Society of Urological Pathology; *N*: number of cases; NCS: not clinically significant; PCa: prostate cancer; PSAD: PSA density; PV: prostate volume; phi: Prostate Health Index; PHID: phi density; SD: standard deviation; tPSA: total PSA; %p2PSA: p2PSA to fPSA ratio. ^∗^*P* < 0.05 for Student's *t*-test or Wilcoxon singed rank test vs. non-PCa cases. ^∗∗^*P* < 0.05 for Pearson's chi-squared test. ^#^*P* < 0.05 for Wilcoxon singed rank test vs. HGPIN.

**Table 2:** Sensitivity, specificity, and positive and negative predictive values of blood serum biomarkers in predicting overall prostate cancer.

	According to Youden's index					90% sensitivity			
	Cut-off	Sensitivity (% (95% CI))	Specificity (% (95% CI))	PPV (% (95% CI))	NPV (% (95% CI))	Cut-off	Specificity (% (95% CI))	PPV (% (95% CI))	NPV (% (95% CI))
tPSA (ng/mL)	4.18	44.6 (35.2-54.3)	62.2 (51.9-71.8)	57.5 (46.4-68.0)	49.6 (40.5-58.8)	2.5	11.2 (5.0-17.5)	53.7 (46.6-60.9)	50.0 (29.1-70.9)
PSAD (ng/mL/cc)	0.09	70.5 (61.2-78.8)	65.3 (55.0-74.6)	69.9 (60.6-78.2)	66.0 (55.7-75.3)	0.05	24.5 (16.4-34.2)	58.0 (50.3-65.3)	70.6 (52.5-84.9)
fPSA (ng/mL)	0.45	37.5 (28.5-47.2)	81.6 (72.5-88.7)	70.0 (56.8-81.2)	53.3 (45.0-61.5)	0.26	3.1 (0.0-6.5)	51.5 (44.5-58.5)	21.4 (0.0-42.9)
%fPSA	11.41	43.8 (34.4-53.4)	89.8 (82.0-95.0)	83.1 (71.0-91.6)	58.3 (50.0-66.2)	7.00	1.0 (0.0-3.0)	50.8 (43.8-57.7)	7.7 (0.0-22.2)
p2PSA (pg/mL)	17.69	17.0 (10.5-25.2)	87.8 (79.6-93.5)	61.3 (42.2-78.2)	48.0 (40.5-55.6)	5.04	1.0 (0.0-3.0)	51.0 (44.1-58.0)	8.3 (0.0-24.0)
%p2PSA	1.77	77.7 (68.8-85.0)	46.9 (36.8-57.3)	62.6 (54.0-70.6)	64.8 (52.5-75.8)	1.41	29.6 (20.8-39.7)	59.4 (51.6-66.9)	72.5 (58.7-86.3)
phi	44.49	56.3 (46.6-65.6)	83.7 (74.8-90.4)	79.7 (69.2-88.0)	62.6 (53.7-70.9)	25.93	23.5 (15.1-31.9)	57.4 (50.1-64.7)	67.6 (51.9-83.4)
PHID	1.04	61.6 (51.9-70.6)	81.6 (72.5-88.7)	79.3 (69.3-87.3)	65.0 (55.9-73.4)	0.54	35.7 (26.3-46.0)	61.8 (53.9-69.3)	77.8 (62.9-88.8)

Abbreviations: CI: confidence interval; free PSA: free prostate-specific antigen; %fPSA: free to total PSA ratio; NPV: negative predictive value; PPV: positive predictive value; PSAD: prostate-specific antigen density; phi: Prostate Health Index; PHID: phi density; tPSA: total PSA; %p2PSA: p2PSA to fPSA ratio.

**Table 3:** Sensitivity, specificity, and positive and negative predictive values of blood serum biomarkers in predicting clinically significant prostate cancer according to Epstein's criteria.

	According to Youden's index					90% sensitivity			
	Cut-off	Sensitivity (% (95% CI))	Specificity (% (95% CI))	PPV (% (95% CI))	NPV (% (95% CI))	Cut-off	Specificity (% (95% CI))	PPV (% (95% CI))	NPV (% (95% CI))
tPSA (ng/mL)	4.28	51.9 (40.5-63.1)	71.3 (62.7-78.9)	53.2 (41.6-64.5)	70.2 (61.6-77.9)	2.77	24.0 (16.7-31.4)	42.7 (35.3-50.1)	79.5 (66.8-92.2)
PSAD (ng/mL/cc)	0.09	81.5 (71.3-89.3)	61.2 (52.3-69.7)	56.9 (47.4-66.1)	84.0 (75.1-90.8)	0.07	41.9 (33.2-50.9)	49.3 (41.0-57.7)	87.1 (76.2-94.3)
fPSA (ng/mL)	0.44	38.3 (27.7-49.7)	82.2 (74.5-88.4)	57.4 (43.2-70.8)	67.9 (60.0-75.2)	0.28	4.7 (1.0-8.3)	36.9 (30.2-43.7)	40.0 (15.2-64.8)
%fPSA	12.15-12.90	59.3 (47.8-70.1)	86.8 (79.7-92.1)	73.8 (61.5-84.0)	77.2 (69.6-83.8)	7.00	1.6 (0.0-3.7)	35.5 (28.9-42.2)	15.4 (0.0-35.0)
p2PSA (pg/mL)	15.19	28.4 (18.9-39.5)	77.5 (69.3-84.4)	44.2 (30.5-58.7)	63.3 (55.3-70.8)	5.80	5.4 (1.5-9.3)	37.4 (30.6-44.2)	46.7 (21.4-71.9)
%p2PSA	2.07	69.1 (57.9-78.9)	64.3 (55.4-72.6)	54.9 (44.7-64.8)	76.9 (67.8-84.4)	1.54	31.8 (23.8-39.8)	45.3 (37.7-53.0)	83.7 (70.3-92.7)
phi	44.47	69.1 (57.9-78.9)	81.4 (73.6-87.7)	70.0 (58.7-79.7)	80.8 (72.9-87.2)	31.92	35.7 (27.4-43.9)	46.8 (39.0-54.6)	85.2 (75.7-94.7)
PHID	1.06	71.6 (60.5-81.1)	78.3 (70.2-85.1)	67.4 (56.5-77.2)	81.5 (73.5-87.9)	0.61	36.4 (28.1-45.4)	47.1 (39.0-55.3)	85.5 (73.3-93.5)

Abbreviations: CI: confidence interval; fPSA: free prostate-specific antigen; NPV: negative predictive value; PPV: positive predictive value; PSAD: prostate-specific antigen density; phi: Prostate Health Index; PHID: phi density; tPSA: total PSA; %fPSA: free to total PSA ratio; %p2PSA: p2PSA to fPSA ratio.

**Table 4:** Sensitivity, specificity, and positive and negative predictive values of blood serum biomarkers in predicting ISUP ≥ 2 grade prostate cancer.

	According to Youden's index					90% sensitivity			
	Cut-off	Sensitivity (% (95% CI))	Specificity (% (95% CI))	PPV (% (95% CI))	NPV (% (95% CI))	Cut-off	Specificity (% (95% CI))	PPV (% (95% CI))	NPV (% (95% CI))
tPSA (ng/mL)	4.48	47.5 (31.5-63.9)	68.2 (60.7-75.2)	26.0 (16.5-37.6)	84.7 (77.5-90.3)	2.50	10.6 (6.0-15.2)	19.1 (13.5-24.8)	81.8 (65.7-97.4)
PSAD (ng/mL/cc)	0.13	57.5 (40.9-73.0)	77.6 (70.1-83.7)	37.7 (25.6-51.0)	88.6 (82.4-93.2)	0.04	5.3 (2.5-9.8)	19.1 (13.9-25.3)	81.8 (48.2-97.7)
fPSA (ng/mL)	0.73	77.5 (61.6-89.2)	34.1 (27.0-41.8)	21.7 (15.2-29.3)	86.6 (76.0-93.7)	0.29	6.5 (2.8-10.2)	18.5 (13.0-23.9)	73.3 (51.0-95.7)
%fPSA	11.30-11.69	57.5 (40.9-73.0)	78.8 (71.9-84.7)	39.0 (26.6-52.6)	88.7 (82.6-93.3)	7.00	4.7 (1.5-7.9)	17.8 (12.4-23.1)	61.5 (35.1-88.0)
p2PSA (pg/mL)	26.11-30.29	7.5 (1.6-20.4)	96.5 (92.5-98.7)	33.3 (7.5-70.1)	81.6 (75.5-86.7)	6.21	8.8 (4.6-13.1)	18.8 (13.3-24.4)	78.9 (60.6-97.3)
%p2PSA	1.88	85.0 (70.2-94.3)	47.6 (39.9-55.4)	27.6 (20.0-36.4)	93.1 (85.6-97.4)	1.65	34.1 (27.0-41.2)	24.3 (17.7-32.1)	93.5 (87.4-100.0)
phi	44.71	75.0 (58.8-87.3)	72.4 (65.0-78.9)	39.0 (28.1-50.8)	92.5 (86.6-96.3)	33.20	34.7 (27.6-41.9)	24.5 (17.54-31.4)	93.7 (87.6-100.0)
PHID	1.04	75.0 (58.8-87.3)	66.5 (58.8-73.5)	34.5 (24.6-45.4)	91.9 (85.6-96.0)	0.63	34.1 (27.0-41.8)	24.3 (17.4-31.2)	93.5 (87.4-98.2)

Abbreviations: CI: confidence interval; fPSA: free prostate-specific antigen; ISUP: International Society of Urological Pathology; NPV: negative predictive value; PPV: positive predictive value; PSAD: prostate-specific antigen density; phi: Prostate Health Index; tPSA: total PSA; %fPSA: free to total PSA ratio; %p2PSA: p2PSA to fPSA ratio.

**Table 5:** Area under the receiver operating characteristic curve for prostate cancer biomarkers and logistic regression models.

Biomarker	Overall PCa	Clinically significant PCa	
		According to Epstein's criteria	ISUP grade ≥ 2
	AUC (95% CI) *P* value	AUC (95% CI) *P* value	AUC (95% CI) *P* value
tPSA (ng/mL)	0.56 (0.48-0.64) 0.119	0.65 (0.57-0.72) 0.000	0.58 (0.48-0.68) 0.110
PSAD (ng/mL/cc)	0.72 (0.65-0.79) 0.000	0.78 (0.72-0.85) 0.000	0.68 (0.59-0.78) 0.000
fPSA (ng/mL)	0.62 (0.55-0.70) 0.002	0.62 (0.54-0.70) 0.003	0.62 (0.52-0.72) 0.003
%fPSA	0.68 (0.61-0.75) 0.000	0.76 (0.69-0.83) 0.000	0.71 (0.63-0.80) 0.000
p2PSA (pg/mL)	0.52 (0.44-0.60) 0.576	0.52 (0.44-0.61) 0.60	0.54 (0.44-0.64) 0.60
%p2PSA	0.68 (0.61-0.75) 0.000	0.70 (0.63-0.77) 0.000	0.72 (0.64-0.80) 0.000
phi	0.72 (0.65-0.79) 0.000	0.77 (0.71-0.84) 0.000	0.77 (0.69-0.84) 0.000
PHID	0.77 (0.70-0.83) 0.000	0.80 (0.74-0.86) 0.000	0.75 (0.66-0.83) 0.000
Base model	0.75 (0.69-0.82) 0.000	0.81 (0.74-0.87) 0.000	0.74 (0.65-0.83) 0.000
Base model +p2PSA	0.78 (0.72-0.84) 0.000	0.83 (0.77-0.89) 0.000	0.78 (0.70-0.86) 0.000
Base model +%p2PSA	0.78 (0.71-0.84) 0.000	0.82 (0.76-0.88) 0.000	0.78 (0.70-0.86) 0.000
Base model +phi	0.78 (0.72-0.84) 0.000	0.83 (0.76-0.89) 0.000	0.79 (0.71-0.87) 0.000
Base model +PHID	0.77 (0.71-0.84) 0.000	0.80 (0.74-0.87) 0.000	0.75 (0.66-0.83) 0.000

Abbreviations: AUC: area under the receiver operating curve; CI: confidence interval; fPSA: free prostate-specific antigen; %fPSA: free to total PSA ratio; ISUP: International Society of Urological Pathology; OR: odds ratio; PCa: prostate cancer; %p2PSA: p2PSA to fPSA ratio; phi: Prostate Health Index; PHID: phi density; PSAD: PSA density; tPSA: total PSA.

**Table 6:** Multivariate logistic regression model analysis predicting the overall and clinically significant prostate cancer.

Predictors	Overall PCa					Clinically significant PCa									
						According to Epstein's criteria					ISUP grade ≥ 2				
	Base model	Base model+p2PSA	Base model+%p2PSA	Base model+phi	Base model+PHID	Base model	Base model+p2PSA	Base model+%p2PSA	Base model+phi	Base model+PHID	Base model	Base model+p2PSA	Base model+%p2PSA	Base model+phi	Base model+PHID
	OR (95% CI) *P* value	OR (95% CI) *P* value	OR (95% CI) *P* value	OR (95% CI) *P* value	OR (95% CI) *P* value	OR (95% CI) *P* value	OR (95% CI) *P* value	OR (95% CI) *P* value	OR (95% CI) *P* value	OR (95% CI) *P* value	OR (95% CI) P value	OR (95% CI) *P* value	OR (95% CI) *P* value	OR (95% CI) *P* value	OR (95% CI) *P* value
Repeated biopsy	0.44	0.34	0.44	0.37	0.37	—	0.36	—	—	—	—	—	—	—	—
	(0.20-0.99)	(0.14-0.80)	(0.19-0.99)	(0.16-0.86)	(0.16-0.86)	—	(0.13-0.98)	—	—	—	—	—	—	—	—
	0.048	0.013	0.048	0.021	0.019	>0.05	0.046	>0.05	>0.05	>0.05	>0.05	>0.05	>0.05	>0.05	>0.05
PV (mL)	0.96	0.96	0.97	0.97	—	0.96	0.96	0.96	0.97	—	—	—	—	—	—
	(0.95-0.98)	(0.95-0.98)	(0.95-0.98)	(0.95 0.99)	—	(0.94-0.98)	(0.94-0.99)	(0.94-0.99)	(0.95-0.99)	—	—	—	—	—	—
	0.000	0.000	0.000	0.001	>0.05	0.000	0.001	0.0012	0.004	>0.05	>0.05	>0.05	>0.05	>0.05	>0.05
fPSA (ng/mL)	—	—	—	—	—	4.38	—	6.59	—	—	—	—	—	—	—
	—	—	—	—	—	(1.22-15.71)	—	(1.68-25.97)	—	—	—	—	—	—	—
	>0.05	>0.05	>0.05	>0.05	>0.05	0.024	>0.05	0.007	>0.05	>0.05	>0.05	>0.05	>0.05	>0.05	>0.05
%fPSA	0.94	0.91	—	—	—	0.85	0.85	0.86	0.93	0.93	0.88	0.84	0.91	0.93	0.93
	(0.90-0.99)	(0.86-0.96)	—	—	—	(0.80-0.92)	(0.79-0.91)	(0.80-0.92)	(0.88-0.99)	(0.88-0.99)	(0.82-0.94)	(0.78-0.91)	(0.85-0.98)	(0.86-1.00)	(0.88-0.99)
	0.022	0.001	>0.05	>0.05	>0.05	0.000	0.000	0.000	0.032	0.031	0.000	<0000	0.009	0.045	0.031
p2PSA (pg/mL)		1.10					1.13					1.09			
		(1.03-1.17)					(1.06-1.22)					(1.03-1.15)			
		0.003					0.000					0.004			
%p2PSA			2.01					1.95					2.11		
			(1.24-3.27)					(1.13-3.35)					(1.27-3.49)		
			0.005					0.016					0.004		
phi				1.05					1.05					1.04	
				(1.02-1.07)					(1.02-1.08)					(1.02-1.07)	
									0.001					0.002	
PHID					4.34					3.58					2.38
					(2.52-7.48)					(2.03-6.30)					(1.61-3.53)
					0.000					0.000					0.000

Abbreviations: CI: confidence interval; fPSA: free prostate-specific antigen; ISUP: International Society of Urological Pathology; OR: odds ratio; PCa: prostate cancer; PV: prostate volume; phi: Prostate Health Index; PHID: phi density; tPSA: total PSA; %fPSA: free to total PSA ratio; %p2PSA: p2PSA to fPSA ratio.

## Data Availability

The data used to support the findings of this study are available from the corresponding author upon request.
